# A Comprehensive Review on Postmenopausal Osteoporosis in Women

**DOI:** 10.7759/cureus.48582

**Published:** 2023-11-09

**Authors:** Samruddhi H Charde, Abhishek Joshi, Juhi Raut

**Affiliations:** 1 Community Medicine, Jawaharlal Nehru Medical College, Datta Meghe Institute of Higher Education and Research (Deemed to be University), Wardha, IND

**Keywords:** management, bone, lifestyle, postmenopause, osteoporosis

## Abstract

In India, a sizeable share of the female population is in the postmenopausal or perimenopausal stage. Issues related to aging in women are an increased risk of broken bones, a decrease in cortical and cancellous bone thickness, and a decrease in bone mineral density (BMD). Osteoporosis has a severely detrimental effect on the life of women, lowering their standard of living, decreasing the quality of their lives, and increasing their likelihood of fractures. It can be terrible if the fracture affects the hip or the spine since it could leave you immobile. Postmenopausal osteoporosis is related to lack of estrogen and lack of eggs produced by ovaries seen with increasing age. After many years of study, the role of estrogen is now well established in bone remodelling. Estrogen contributes to the resorption and strengthening of bone. It detects less density of bone at specific site and helps in strengthening the bone at that location. Treatment choices are based on severity, rate of advancement, and individual patient-specific characteristics. By adopting a lifecycle approach, all women should be educated about this illness and inspired to adopt a healthy lifestyle that includes regular exercise and a balanced diet. All premenopausal women should be advised to take vitamin D and calcium supplements regardless of whether or not they have any bone defects. Smoking and alcohol consumption should also be restricted. Pharmacological intervention is carried out on patients diagnosed with the disease. Drugs should be chosen based on their side effects and contradictions. It's crucial to do follow-up, and patient compliance should be closely observed. This article raises awareness of this widespread illness and helps women of postmenopausal age take the required precautions to stop bone thinning and manage its progress; moreover, it also reviews the literature already published on it.

## Introduction and background

As people age, the need to control and avoid osteoporotic fragility fractures increases. Bone density loss and microstructure tissue bone deterioration are symptoms of the skeleton-related disorder called osteoporosis. Osteoporosis, which means "porous bone", makes bones more brittle and susceptible to fractures. Its treatment has had many advances in the last 50 years, which was once thought to be an inevitable side effect of aging. This includes the widespread availability of various potent pharmacological medications [1]. In men, testosterone is crucial for bone health, while in women, estrogen plays an important role. Men have stronger bones than women and experience less lifetime bone loss. In addition, men experience fractures less frequently than women while having a greater death rate after a fracture. Secondary osteoporosis is more common in men than in women [2]. According to studies, bone loss begins in men and women between the ages of 30 and 40. There has been speculation that within a year after menopause in women, bone mass and density may decline. About 10 years following menopause, this accelerated rate of bone loss achieves an equilibrium and then combines with an ongoing aging-related loss of muscle mass [3].

Thanks to the Study of Women's Health Across the Nation (SWAN), we now have a far better grasp of how the female's bone health alters during menopausal transition (MT), expanding our awareness of a crucial period that has a significant impact on osteoporosis risk as people get older. One of the key sources for the review was the said study which provided extensive longitudinal assessments of bone health over MT [4].

After the age of 50, loss of muscle mass is significant along with similar gender-neutral changes like increased satellite cell senescence and inflammation, decreased protein synthesis and myocyte regeneration, and several other gender-specific changes caused by the depletion of sex hormones [5]. Sarcopenia affects both sexes due to the decline in testosterone in males and estrogen in women [5]. Numerous studies have shown a link between other age-related conditions and osteoporosis marked by fracture risk, the decreasing density of mineral and bone, bone tissue frailty, and sarcopenia, and the presence of fragility factors or the bone mineral density (BMD) criteria is used to diagnose osteoporosis. Older people have more chances of developing osteoporosis, which affects women more frequently and often starts around menopause [5].

## Review

Methodology

**Figure 1:**
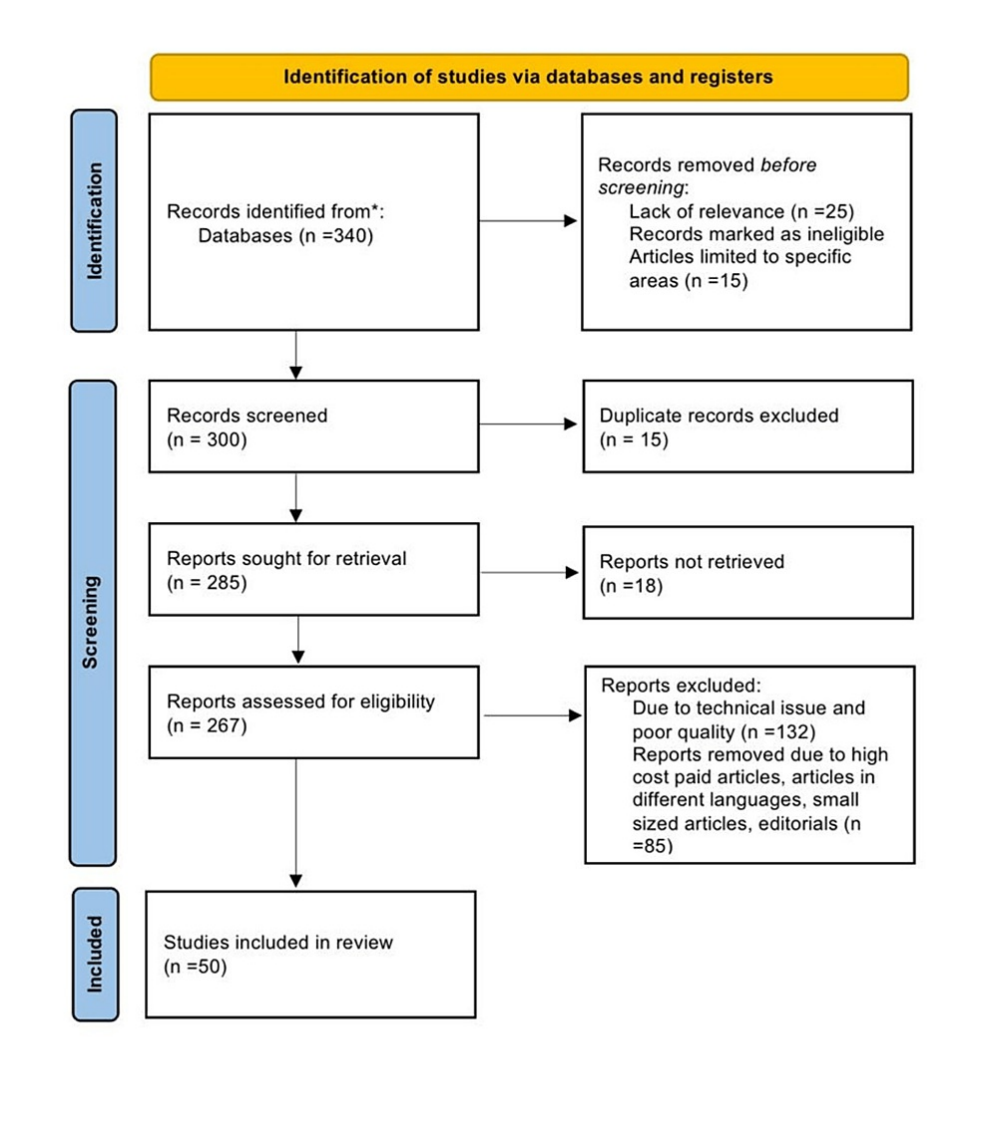
PRISMA methodology PRISMA: Preferred Reporting Items for Systematic Reviews and Meta-Analysis

Using the electronic databases ResearchGate, Google Scholar, Embase, MEDLINE, and PubMed, a search of the English-language literature was done. It was also the subject of a different search. The query terms were "postmenopausal", "etiology" OR "causes", "osteoporosis" OR "bone loss", and "lifestyle changes" OR "management". The following requirements are met in this review article: studies conducted exclusively on postmenopausal bone loss and its management and studies that were conducted 10 years ago in English. Figure 1 highlights the Preferred Reporting Items for Systematic Reviews and Meta-Analysis (PRISMA) method used in this research methodology.

Osteoporosis

Osteoporosis is a condition which is characterized by the disruption of bone microarchitecture, bone tissue deterioration, and low bone mass: it can lead to compromised bone strength and an increased risk of fractures [1]. It is a crippling ailment having noticeable adverse effects on the body, the mind, the society, and the economy [6]. The process of aging naturally brings the primary osteoporosis condition, and some systemic diseases and clinical pathologies cause secondary osteoporosis [7]. Estrogen insufficiency is linked to osteoporosis in postmenopausal age. This deficit causes growth in osteoclast activity and manufacture, which weakens the bone trabeculae and increases the fracture risk. It is apparent that estrogen negatively affects osteoclast production and function since estrogen replacement therapy counteracts these effects, although it is unknown how estrogen achieves this [8].

Bone Remodelling

An array of mechanisms supporting osteoblastogenesis and osteoclastogenesis maintains bone, which consists of mineralized matrix which makes it a connective body structure. Bone is a composite structure that manages numerous functions, including maintaining skeletal size, maintaining form integrity, housing marrow, and regulating mineral homeostasis. Bone development and maintenance are based on modelling and remodelling [9]. The dynamic and lifelong procedure of bone remodelling preserves bone quality and mass, prevents the buildup of hypermineralized bone, and regulates mineral homeostasis by releasing reserves of phosphorus and calcium ions (Ca2+). The osteoclasts do the resorption, and in a new bone production, osteoblasts are involved; these two processes are tightly coupled during the process of bone turnover [10].

Five key steps are included in the bone remodelling cycle: activation, reversal, resorption, termination, and formation. The skeleton remodels itself in order to regulate maintenance and repair, and this process provides a way to quickly access calcium and phosphate in order to maintain mineral homeostasis. Frost was the first to describe the bone remodelling cycle, which replaces damaged and old bone with new bone [11]. Osteoblasts must repair the bone that osteoclasts have resorbed for bone remodelling to occur correctly. The cellular processes that link resorption to rebuilding remained unexplored for ages. As a result, the remodelling cycle is only partially understood, accounting for only the resorption and creation processes. Recently, it has been cleared by the invention of newer techniques that enable us to understand the remodelling process [12].

Role of estrogen

Some papers advocate the use of menopausal hormone treatment (MHT) for preserving bone health and avoiding impending fracture in women who have recently reached menopause. MHT may function in treating postmenopausal osteoporosis shortly after menopause, despite disagreement regarding related adverse effects that have restricted its usage in recent years [13]. Some research indicates that miR-143/145 has a dual influence on bone resorption and formation and plays both a canonical and noncanonical role in modulating bone marrow stromal cell (BMSC) pluripotency. These findings point to the target therapeutic dose for treating bone loss due to estrogen deficiency [14]. Over the past few decades, prescribing guidelines for MHT have changed according to the known advantages and disadvantages of the treatment. Previously, MHT was widely recognized as safe and appropriate in the management of menopausal symptoms and for lowering the risk of chronic illnesses including cardiovascular disease (CVD) [15]. Losses of bone and skeletal muscle were related to MT. For skeletal muscle, increased physical activity during various menopausal periods proved advantageous. More commonly developed conditions in women are sarcopenia, fractures, osteoporosis, and mobility impairment from falls later due to menopause-related hormonal changes. To encourage physical exercise among middle-aged women, new tactics are required [16].

A natural anti-oxidant having anti-oxidative and anti-aging properties is pyrroloquinoline quinone (PQQ). However, it is unclear if PQQ protects against osteoporosis brought on by a deficiency in estrogen. In this work, using a mouse model of osteoporosis caused by ovariectomy, we assessed PQQ's effects on biomechanical strength, bone turnover, and bone microarchitecture. Regarding its ability to stop bone loss induced by ovariectomy and boost bone strength, PQQ supplementation was comparable to exogenous estrogen therapy. It did this by promoting osteoblastic differentiation by reducing osteocyte senescence, oxidative stress, and the senescence-associated secretory phenotype (SASP). This was true even though dietary PQQ supplementation did not affect the uterine weight or serum estrogen levels of ovariectomy mice [17]. Hence, MHT ought to be considered as a possible alternative for maintaining women's skeletal health, specifically as an added benefit in treating menopausal symptoms, whether started shortly after or at menopause, with the framework of a customized benefit and risk analysis [13].

Risk factors

Patients with previously fractured osteoporosis can frequently experience forearm, ankle, and foot fractures. It is contended that it's critical to identify osteoporosis before the first fracture occurs and to carry out a disease-specific quality-of-life assessment [18].

The epidemiology of fractures is influenced by race and ethnicity; the greatest fracture rate is in white women. Despite having a higher BMD, African American women stay longer in hospitals, are less likely to be mobile after being discharged, and most probably die from fractures of the hip. Decreased BMD, older age, a fracture in the past, and a history of falls more than two times are risk factors for fracture across all ethnic groups. Depending on one's race and ethnicity, osteoporosis is detected, diagnosed, and treated differently. Asian women and white women are more prone to it than black women [19].

Osteoporosis has a complicated etiology, and hereditary factors may be responsible for up to 50-85% of postmenopausal women's chance of developing the illness. The WNT16 gene's polymorphism seems to be highly related to the pathophysiology of osteoporosis, making it an attractive target for further investigation into the genetic causes of fracture risk [20].

According to some studies, the standalone risk factor for osteoporosis in women in postmenopausal age is smoking. Loss of bone mass can be stopped by including physical exercise in one's daily routine. It is essential to emphasize the significance of these factors on bone health from an early age through education and specific preventive interventions [21]. Apart from menopause, other factors like ovariectomy, smoking, having a lean body type, not exercising, getting insufficient calcium, and consuming too much animal protein, phosphorus, sodium, caffeine, and alcohol also increase the risk of developing osteoporosis. Certain minerals, among other nutrients, are related to bone density. The connection between postmenopausal women's eating behaviors and bone density has been investigated in some research. The results revealed a link between bone density and blood minerals such as calcium, phosphorus, magnesium, copper, zinc, and manganese [22].

Personal habits such as chronic cigarette smoking, binge alcohol consumption (two or more drinks on most days), a high level of caffeine consumption, insufficient intake of milk and its derivatives which can lead to dietary calcium deficiency, inactivity or increase in sedentary behavior, excessive consumption of saturated fatty acids, and a reduced intake of monounsaturated fatty acids (MUFA) and vitamin D deficiency due to lack of sunlight are likely to cause osteoporosis [22].

Diagnosis

The primary technique for identifying osteoporosis is bone densitometry, which analyzes the mineral content and density of bone. It can be carried out with X-rays, in which case it is referred to as dual-energy X-ray absorptiometry (DEXA) or DXA, or with CT using a software that scans the hip and spine to calculate BMD [23]. The gold standard is DEXA. Bone densitometry can predict the likelihood of fractures in the future and discover deteriorating bones early enough to benefit from timely treatment [24].

A standard deviation of 2.5 or more below the mean T score for young adults is the criteria used by the World Health Organization. The T score grade for normal bone density is between +1 and -1, for osteopenia between -1 and -2.5, and for osteoporosis beyond -2.5 [23].

The standard deviation unit is calculated from the average value of BMD that is predicted in adults of young age [25]. Another scale that determines units of standard deviation is the Z score, which is typically applied to children and adolescents. It is determined similar to how T scores are determined, but comparisons are performed with people who share the subject's sex, age, weight, height, and race [23].

Management

General Management

It includes dietary changes, frequent exercise, abstaining from vices like drinking alcohol and smoking, taking vitamin D and calcium supplements as directed by the doctor, and avoidance of falling so as to prevent fractures and other injuries. Adopting a lifecycle approach, all women of different age groups are advised to keep a balanced nutrition of proteins and calcium. Most recent recommendations from the National Osteoporosis Foundation (NOF) call for women to consume 1200 milligrams of calcium daily [10]. A sufficient intake of calcium supports bone health, mediates muscle and blood vessel contractions, transmits nerve impulses, and participates in intracellular and extracellular signalling. Calcium is a vital component of mineralized tissues in the body [26].

In women's life, MT is a crucial time. Exercise is considered the most efficient non-pharmaceutical strategy to manage the risk factors linked to the significant estrogen reduction throughout the perimenopausal and early postmenopausal period [27]. A prior fragility fracture with an uncertain recency is associated with a severe osteoporotic fracture with a 10-year risk of 16%. History of a hip fracture in the family gives indications to make lifestyle modifications. A highly risky situation can require an anabolic program [28].

Age-related bone loss and muscle loss can be avoided by eating enough protein, whether from dairy or plants. For postmenopausal women, the recommended dietary allowance (RDA) for protein is 1.1-1.2 g/kg/day [29]. At multiple sites, higher intakes of nuts, whole grains, and fruits and lower intakes of processed meat, saturated fats, and sugar are linked to higher BMD [30]. Risk factors for postmenopausal osteoporosis include immobility and extended inactivity. Exercise, including weight-bearing, can increase bone mass and lower fracture risk. Walking leads to postural stability and an increase in hip BMD [31-33]. A study by Roghani et al. discovered that ladies wearing weighted vests while walking had better balance than those who weren't [34]. For those who have lost bone mass, a mix of resistance training and weight-bearing exercise is compelling. This improves spinal and hip bone density as well as functional performance. Yoga can improve posture and build bone density when practiced under a trainer's guidance [35,36].

First-line therapies for postmenopausal osteoporosis include vitamin D and calcium supplements. It is advised to deliver calcium citrate maleate to patients using medications that lower gastric acid secretion and to patients not in good nutritional habits because acid is necessary to absorb calcium carbonate. In India, a lack of vitamin D affects over 70% of the population. Cholecalciferol is a vitamin D supplement that should be taken by all women of postmenopausal age, regardless of whether or not they have osteoporosis. It is advised to be used in doses of 60,000 units once every one to two months. Rarely, if ever, is calcitriol or active vitamin D administered without renal impairment [37].

Pharmacological Management

Pharmaceutical intervention is typically only considered in high-risk circumstances, especially in postmenopausal women with BMDs that correspond to a T score of 2.5 [10].

What Is Fracture Risk Assessment Tool (FRAX)?

FRAX is a tool doctors usually use to estimate an individual's 10-year probability of incurring a hip or other major osteoporotic fracture [38]. A person's age, weight, gender, smoking history, alcohol consumption, fracture history, history of glucocorticoid use (which can promote bone loss), and estimated BMD and the presence of concurrent diseases like rheumatoid arthritis are all taken into consideration when assessing their risk. The results of the aforementioned questions are then used to create a score that generates a 10-year risk of hip fracture as well as a 10-year risk% for any major fracture that may be caused by osteoporosis. Even in younger patients, a score of <5 necessitates a doctor's attention and can call for therapy. At age 70 and higher, a score of >5 for a hip fracture denotes the need for modifications in daily life and pharmaceutical treatment [39].

FRAX has some restrictions in use, as some knowledge about its use with people who didn’t received any osteoporosis medication is not known. It also does not mention the dose of medication or treatment for a person with multiple fractures. It disregards other regions, such as the lumbar vertebrae, and exclusively calculates the BMD of the femoral neck. It also neglects to mention how much and for how long glucocorticoids are used [40,41].

Pharmacological therapy can be broadly divided into three categories: anabolic drugs, estrogen-based hormone replacement therapy (HRT), and anti-resorptive drug therapy. Estrogen is a unique substance that affects the bone in both anabolic and anti-resorptive ways. Examples of anti-resorptive drugs are denosumab and bisphosphonates. Anabolic agents assist in achieving the same goal by promoting the creation of a new bone, while anti-resorptive drugs lessen the risk of fractures by reducing bone loss [42].

Estrogen Therapy

It seems fair to examine estrogen as a treatment option in both postmenopausal and perimenopausal women because the postmenopausal osteoporosis pathophysiology is well recognized. A research done by the Women's Health Initiative in 2002 revealed that utilizing estrogen has numerous adverse effects that outweigh any positive aspects. It discovered that taking estrogen for HRT for five years or longer increased the risk of coronary heart disease (CHD), venous thromboembolism, stroke, and breast cancer. Cholecystitis was also a potential side effect [43].

These results were later amended to better understand the disadvantages and advantages of MHT. When a woman has early menopause or primary ovarian insufficiency, which results in an estrogen shortage, estrogen treatment has substantial advantages. Women undergoing surgical menopause may benefit from MHT to alleviate further excruciating vasomotor symptoms. Additionally, it can treat genitourinary issues such vulval itching, vaginal dryness, nocturia, urge incontinence, dyspareunia, frequent urination, and urinary tract infections (UTIs) [44].

The forearm, lumbar vertebrae, and femoral neck are just a few of the places in postmenopausal women where HRT was discovered to maintain and even increase BMD. Estrogen medication can be started in postmenopausal females under 60 who are at risk for fractures or are within 10 years after menopause as a first line of treatment; however, estrogen therapy is not recommended in women over 60 due to the risk of developing breast cancer. Progesterone is advised for use in conjunction with estrogen in women who still have their uteruses, as long-term usage of unopposed estrogen can result in endometrial hyperplasia and cancer. Estrogen can be administered alone to females without uteri [10,44].

Anti-resorptive Agents

According to a research, zoledronic acid infusions of 5 mg once a year throughout a three-year period significantly reduced the incidence of fracture at all major osteoporotic fracture sites, including the two main endpoints of hip and vertebral fractures. The decreased risk of vertebral fracture rates of 70% was more significant than the reductions in fracture rates linked to other anti-resorptive drugs and the 40-50% reduction in oral bisphosphonate fracture rates found during three years. Clinical vertebral fractures and all different prospectively defined categories of fractures, including non-vertebral fractures, were also considerably decreased [45].

Alendronate treatment for four years enhanced BMD in women with poor BMD who had no vertebral fractures while lowering the risk of their first vertebral deformity. For women with osteoporosis but not for those with higher BMD, alendronate dramatically decreased the likelihood of clinical fractures [46].

Ibandronate reduces the incidence of vertebral fractures by 50% over three years, and data suggest its effectiveness in lowering non-vertebral fractures. Some of its side effects are stomach ulcers, heartburn, esophageal ulcers, allergic reactions, and painful bones, muscles, or joints. Contraindications include several kidney disorders, achalasia cardia, Barrett's esophagus, gastritis, hypocalcemia, and bedridden patient in case of invasive dental procedures. The dose is 150 mg per month [47].

Risedronate reduces the chances of non-vertebral fracture by 33-39% and vertebral fractures by 41-49% with prior fractures. Side effects are stomach and esophageal ulcers, allergic reactions, heartburn, and painful bones, muscles, or joints. Contraindications are gastritis, hypocalcemia, esophageal ulcer, inflammation in the tissues surrounding the tooth, achalasia cardia, and patients undergoing invasive dental procedures. The dose to be taken is 35 mg per week [48].

A human monoclonal antibody called denosumab binds to and suppresses the cytokine receptor activator of nuclear factor kappa-Β ligand (RANKL). It decreases bone resorption by preventing osteoclasts from maturing and surviving. It may be given to CKD patients. Subcutaneous injections are frequently administered once every six months. Hypocalcemia, hypersensitivity responses, and excessive inhibition of bone turnover are some of denosumab's most frequent adverse effects. It has been discovered that using denosumab results in a 68% drop in vertebral fractures and a reduction in hip fractures of up to 40% [49].

Calcitonin is a polypeptide that decreases bone turnover by metabolizing calcium and phosphorus. However, it is not recommended as a treatment for osteoporosis due to the cancer risk that comes with long-term usage [50].

Anabolic Agents

Among them is teriparatide. The bioactive component of teriparatide, a synthetic version of parathyroid hormone (PTH), has 34 amino acids. Albright et al. noted in 1934 that PTH was in charge of bone production as well as osteoclasts' breakdown of bone. It was later revealed that PTH exhibited a net anabolic impact on the bone and might thus be helpful in osteoporosis when administered continuously in tiny dosages over an extended period. The results of these studies and further clinical trials later revealed that teriparatide reduced the risk of non-vertebral fractures by 53% and the incidence of vertebral fractures by 65% at an FDA-approved dosage of 20 g/day. In animal studies, teriparatide has been related to an increased risk of osteosarcoma; however, human studies have not indicated a higher incidence. It is disputed in cases of pregnant women, pediatric patients, individuals with Paget's disease, patients with other metabolic bone illnesses besides osteoporosis, patients with pre-existing hypercalcemia, bone cancer patients, and patients with a history of bone radiation therapy. Abaloparatide, a PTH-related peptide analog that has received FDA approval, may have certain benefits over teriparatide [51].

**Figure 2:**
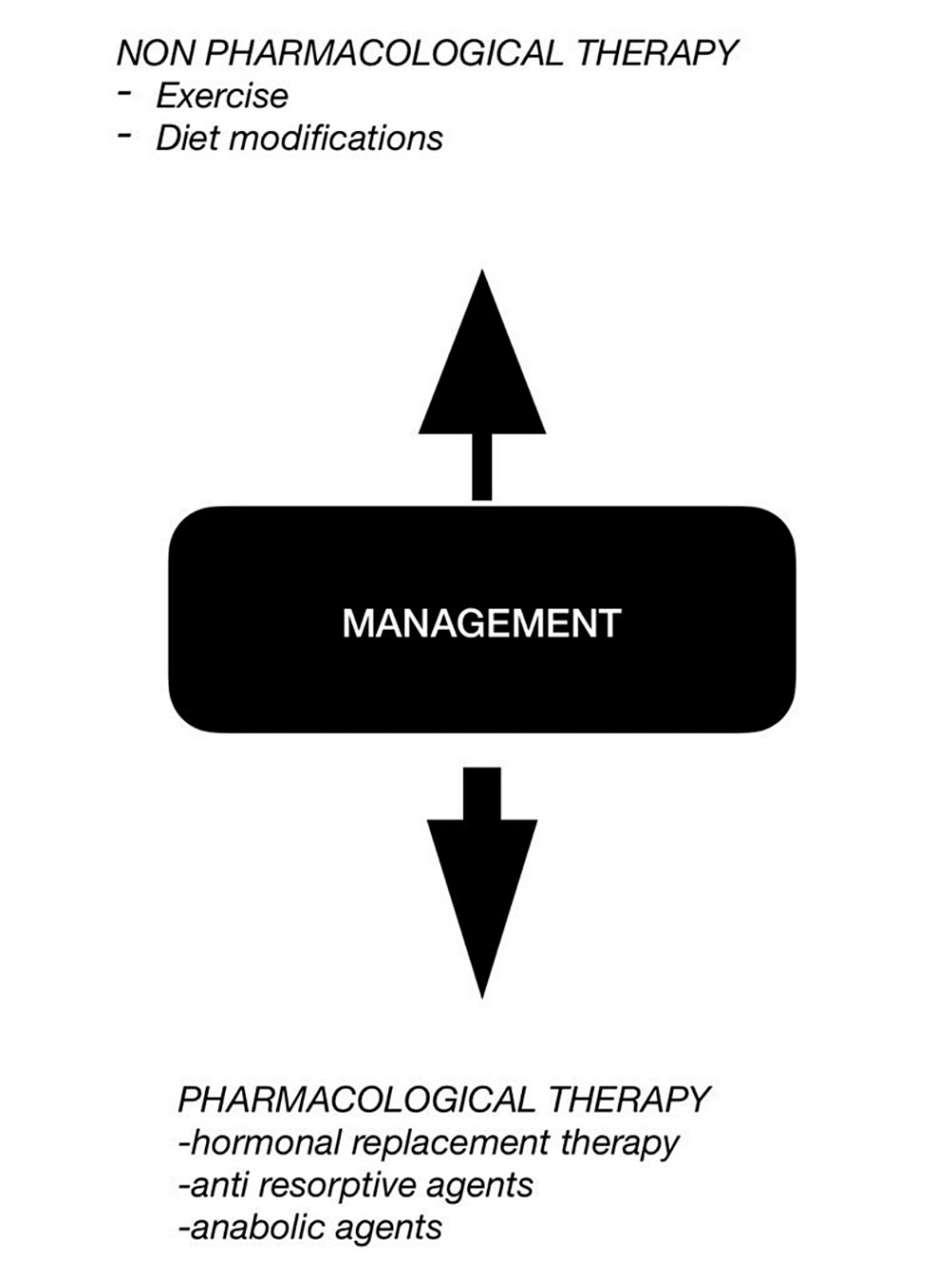
Management modalities of postmenopausal osteoporosis

Figure 2 depicts a summary of the management of postmenopausal osteoporosis.

Findings from multiple studies included in this review are listed in Table 1. 

**Table 1:** Summary table of findings from various sources included in this review in tabulated format along with the year of publication and country of origin

Authors	Year	Country	Findings
Clynes et al. [1]	2020	UK	Very few people suffering from osteoporosis are being treated so focus should be on identifying and treating people who are suffering from it.
Vilaca et al. [2]	2022	USA	Osteoporosis is uncommon among men, but with aging, they should also be aware so as to prevent the disorder.
Kadam et al. [3]	2018	India	Osteoporosis prevalence among men at the lumbar site was lower than postmenopausal women but higher than premenopausal women.
Karlamangla et al. [4]	2018	USA	Low trauma fracture incidence is more in white women than Asian women.
Agostini et al. [5]	2018	Italy	Consumption of protein and vitamin D supplements together with protocols of exercise training is a safe and cheap substitute to estrogen replacement therapy.
NIH Consensus Development Panel on Osteoporosis Prevention, Diagnosis, and Therapy [6]	2001	USA	Attention should be given to skeletal health and proper nutrition; exercise should done regularly for prevention.
Fitzpatrick [7]	2002	USA	Secondary osteoporosis causes can include hypogonadism, medication, hyperthyroidism, vitamin D deficiency, hematological disease, etc.
Hughes et al. [8]	1996	USA	Lack of estrogen causes osteoclast to proliferate and become more active; this punctures the bone trabeculae making them weak and increasing the risk of fracture.
Ramesh et al. [9]	2021	India	Flavonoids have anti-oxidant properties that are associated with the formation of bone and inhibit the resorption of bone.
Bhatnagar and Kekatpure [10]	2022	India	Nowadays, medical and non-medical methods are available for patients. Proper awareness about the prevention of the disorder can help to diagnose and treat people of India effectively.
Kenkre and Bassett [11]	2018	UK	Osteoclastic bone resorption followed by osteoblastic bone formation is coupled to ensure that bone mass is ultimately preserved.
Delaisse et al. [12]	2020	Denmark	At the beginning of the remodelling, catabolic osteoblast lineage cells with low cell density are detected in close proximity to osteoclasts, which promotes osteoclastic resorption.
Rozenberg et al. [13]	2020	Belgium	Menopausal hormone therapy is found to be beneficial in treating symptoms of menopausal osteoporosis.
Xu et al. [14]	2021	China	One potential therapeutic target for the treatment of estrogen-deficient bone loss is miR-143/145.
Mehta et al. [15]	2021	USA	For vasomotor symptoms of menopause, hormonal therapy is the most effective.
Sipilä et al. [16]	2020	Finland	As menopausal transition occurs, bone and skeletal muscle losses are linked to it.
Geng et al. [17]	2019	China	By reducing oxidative stress, osteocyte aging, osteoclastic bone resorption, and promoting osteoblastic bone production, estrogen supplementation can treat osteoporosis caused by estrogen deficiency.
Kuru et al. [18]	2014	Turkey	Patients with osteoporosis who have a history of fractures are frequently encountered with foot, ankle, and forearm fractures.
Cauley [19]	2011	USA	Higher fracture rates are seen in white women.
Mitek et al. [20]	2019	Poland	In the pathogenesis of osteoporosis, polymorphism of WNT16 gene seems highly relevant.
Bijelic et al. [21]	2017	Bosnia and Herzegovina	From the study, the result shows that one independent risk factor for osteoporosis is smoking.
Kim et al. [22]	2008	South Korea	Independent risk factors for osteoporosis are diets rich in total protein, animal protein, or sodium. Low intake of iron, vegetable protein, and calcium appears to be protective.
Kanis et al. [23]	2019	UK	Bone mineral density helps in assessing fracture risk.
Kanis et al. [24]	2019	UK	By preventive measures, this disorder is preventable.
Ferrari et al. [25]	2012	Switzerland	In young adults with low bone density, there are chances of fracture from low-impact trauma, usually in those having bone metabolism disorder.
Vannucci et al. [26]	2018	Italy	A good source of bioavailable calcium is calcium-rich mineral water, which contributes to achieving the daily requirements.
Hettchen et al. [27]	2021	Germany	Positive results are seen after high-intensity exercises in postmenopausal osteoporotic women.
Kanis et al. [28]	2020	UK	Guide for assessing low-, high-, and very-high-risk fracture is provided.
Gregorio et al. [29]	2014	USA	When comparing the group with higher protein consumption to that with reduced protein intake, those with lower intake had lower and upper extremity impairments.
Chen et al. [30]	2016	China	Better adherence to Mediterranean diet was associated with good bone mineral density in elderly and middle-aged Chinese.
Ma et al. [31]	2013	China	As an exercise therapy, walking is not singly beneficial.
Martyn-St James and Carroll [32]	2008	UK	Walking on a daily basis had no noticeable effect on the maintenance of bone mineral density at the spine.
Gába et al. [33]	2016	Czech Republic	Brisk walk reduces the risk of falls.
Roghani et al. [34]	2013	Islamic Republic of Iran	Wearing a weighted vest while exercising helps to improve balance.
Zhao et al. [35]	2015	China	Effectiveness is seen in combined resistance exercise protocols in preserving bone mineral density.
Motorwala et al. [36]	2016	India	Yoga in integrated form is a beneficial and safe mode of physical activity.
G and Gupta [37]	2014	USA	The need for improving vitamin deficiency in the Indian population is important and urgent.
Kanis [38]	2008	UK	A tool has been developed to evaluate the risk of fracture.
Unnanuntana et al. [39]	2010	USA	Bone mineral density with clinical factor association will help in the prediction of fracture risk.
LeBoff et al. [40]	2022	USA	Healthcare provider has a crucial role in making awareness and the early avoidance of osteoporosis causing fracture.
Kanis et al. [41]	2009	UK	FRAX is an algorithm used for assessing fracture probability, which is very beneficial.
Black and Rosen [42]	2016	USA	Pharmacology therapy said to be beneficial is of three categories: anabolic drugs, estrogen-based hormone therapy, and anti-resorptive drug therapy.
Nelson et al. [43]	2002	Poland	Side effects of hormonal therapy include stroke, thromboembolic events, breast cancer, cholecystitis, etc.
Yong and Logan [44]	2021	Singapore	There are more benefits in hormonal therapy so it should be encouraged.
Black et al. [45]	2007	USA	The risk of hip, vertebral, and other fractures is greatly decreased by receiving zoledronic acid infusions once a year for a duration of three years.
Cummings et al. [46]	1998	USA	In women with osteoporosis, alendronate dramatically decreased the likelihood of clinical fractures.
Chesnut III et al. [47]	2004	USA	A practical and efficient substitute for bisphosphonate treatments is oral ibandronate.
Reginster et al. [48]	2000	Belgium	Risedronate helps in improving bone density and reducing the incidence of vertebral fracture.
Cummings et al. [49]	2009	USA	Denosumab administered twice a year subcutaneously for a duration of 36 months was proved to be beneficial in the reduction of fractures.
Khosla and Hofbauer [50]	2017	USA	Patient acceptability towards the drugs available needs to be modified; awareness is needed.
Bodenner et al. [51]	2007	USA	Teriparatide is well tolerated but with few side effects.

## Conclusions

In India and the rest of the globe, postmenopausal osteoporosis is an exceedingly common silent illness. It is a chronic, asymptomatic illness that advances slowly. We have finally identified its pathophysiology and etiology after many years of investigation. Its risk factors are apparent, and its diagnostic equipment is now frequently employed. Postmenopausal osteoporosis may be crippling for people with it, but it is treatable with pharmaceutical and non-pharmacological methods. It can result in a significant decline in quality of life if correct diagnosis and treatment are not received. In order to prevent this illness, it is imperative that all postmenopausal women in India are informed about the lifestyle modifications that are necessary throughout the perimenopausal and postmenopausal stage and that all patients have access to reasonably priced treatment alternatives.
